# Declining Bariatric Surgery Volumes and Shifting Practice Patterns: A Five-Year Analysis of Over One Million Procedures

**DOI:** 10.1007/s11695-026-08795-y

**Published:** 2026-07-15

**Authors:** Mélissa V. Wills, Xinlei Zhu, Doua Elamin, Ricard Corcelles, Matthew Kroh, Jerry Dang, Andrew Strong, Salvador Navarrete, Valentin Mocanu

**Affiliations:** Lerner College of Medicine of Case Western Reserve University, Cleveland Clinic, Cleveland, USA

## Abstract

**Mini Abstract:**

Bariatric surgery volumes are declining despite rising obesity prevalence. Practice patterns have shifted toward more complex conversion procedures and away from sleeve gastrectomy. Quality improvements at accredited centers have maintained low complication rates despite increasing patient complexity, but falling volumes threaten training capacity and access.

**Objective:**

To characterize trends in bariatric surgery volumes, patient complexity, procedure selection, and outcomes from 2020 to 2024.

**Summary Background Data:**

Bariatric surgery remains the most effective obesity treatment, yet fewer than 1% of eligible patients undergo surgery. The COVID-19 pandemic and glucagon-like peptide-1 receptor agonists have disrupted surgical volumes. Understanding current practice patterns is critical for resource planning, fellowship training, and maintaining surgical access as obesity prevalence rises.

**Methods:**

Retrospective analysis of the Metabolic and Bariatric Surgery Accreditation and Quality Improvement Program database (2020 to 2024). Demographics, comorbidities, procedure types, and 30-day outcomes were analyzed. Trends were evaluated using chi-square and linear regression.

**Results:**

Among 1,006,270 procedures, volumes declined 23% from the 2022 peak (230,707 to 177,789 in 2024). Primary procedures decreased from 90.2% to 83.9% of total cases, while conversions increased from 8.9% to 11.0%. Sleeve gastrectomy declined from 73.4% to 70.6% of primary procedures; Roux-en-Y gastric bypass increased from 26.6% to 29.4% (all p<0.0001). Patient complexity increased: ASA class III or higher rose from 79.8% to 82.5%. Despite this, serious complications declined from 3.39% to 3.10% and mortality remained stable (0.08% to 0.06%). Readmissions increased from 3.2% to 3.6%.

**Conclusions:**

Bariatric surgery volumes declined substantially to pandemic levels while patient complexity increased. Conversion procedures now constitute over 11% of all cases. Improving outcomes despite rising complexity reflect quality improvements at accredited centers. Volume decline threatens training capacity and surgical access for the growing obesity epidemic.

**Supplementary Information:**

The online version contains supplementary material available at 10.1007/s11695-026-08795-y.

## Introduction

Bariatric surgery has evolved from its origins in the 1950s to become the most effective intervention for obesity and its associated comorbidities, consistently demonstrating superior outcomes compared to lifestyle and medical management [1]. The 1991 National Institutes of Health Consensus Conference established practice standards and recognized bariatric procedures as safe and effective treatments, helping to transform bariatric surgery from a niche intervention into a mainstream surgical specialty [2, 3]. Since then, procedural volumes have increased from approximately 8,600 in 1993 to 256,000 by 2019 [4]. Despite these improvements and growth, only approximately 1% of eligible patients undergo bariatric surgery, a treatment gap that continues to widen even as obesity prevalence rises [5].

Several factors influence current bariatric surgery practice. Procedure selection continues to evolve as evidence supports the superior long-term metabolic and reflux outcomes of Roux-en-Y gastric bypass and as expanding indications increase patient complexity beyond traditional weight-based criteria [6–8]. External factors like the COVID-19 pandemic and anti-obesity medications have also dramatically affected surgical volumes, while centralization to accredited centers, accelerated post-surgical discharge pathways, and greater surgical experience have improved perioperative safety [9]. These circumstances and improvements may create surprising outcomes: safety may improve despite increasing patient complexity, while shorter hospital stays may lead to changes in readmission patterns.

In the face of these important developments, analyses of contemporary bariatric surgery practice patterns are limited, especially in the post-pandemic period. The emergence of anti-obesity medications has generated considerable apprehension regarding their potential impact on bariatric surgery volumes, but concrete data on procedural trends across primary, revisional, and conversion procedures, patient demographics and comorbidity profiles, procedure selection patterns, and perioperative outcomes remain sparse. Understanding these trends is needed to determine the causes of volume changes, evaluating safety as patient complexity increases, informing resource allocation, and identifying barriers to access. This study analyzes five-year trends in bariatric surgery practice patterns and outcomes using the Metabolic and Bariatric Surgery Accreditation and Quality Improvement Program (MBSAQIP) database from 2020 to 2024.

## Methods

### Study Design and Ethical Approvals

A retrospective analysis of the Metabolic and Bariatric Surgery Accreditation and Quality Improvement Program (MBSAQIP) data registry was performed. Data from 2020 to 2024 were included to capture trends in bariatric surgery practice patterns. The MBSAQIP currently captures clinical data from 902 accredited American and Canadian metabolic and bariatric surgery centers. This database prospectively collects standardized pre-, intra-, and postoperative variables specific to bariatric surgery patients. This study was deemed exempt by the Institutional Review Board due to the anonymity of the data.

## Patient Variables

Basic demographic data including age, sex, race, and body mass index (BMI) were collected. Patient comorbidities included the following: American Society of Anesthesiologists (ASA) classification, tobacco use, type 2 diabetes mellitus (insulin-dependent and non-insulin dependent), hypertension, gastroesophageal reflux disease (GERD), sleep apnea, hyperlipidemia, renal insufficiency, dialysis dependency, history of venous thromboembolism (VTE), venous stasis, preoperative therapeutic anticoagulation, history of myocardial infarction (MI), prior cardiac intervention, and prior percutaneous coronary intervention (PCI). Immunosuppressive therapy use was also captured.

## Operative Characteristics

Bariatric procedures were categorized as primary procedures, conversion procedures, or revision procedures, based on accepted nomenclature from ASMBS. Primary procedures included sleeve gastrectomy (SG), Roux-en-Y gastric bypass (RYGB), endoscopic sleeve gastroplasty (ESG), intragastric balloon placement, biliopancreatic diversion (BPD), biliopancreatic diversion with duodenal switch (BPD-DS), single anastomosis duodeno-ileal bypass with sleeve gastrectomy (SADI-DS), one-anastomosis gastric bypass (OAGB), and other unlisted procedures. Conversion procedures involve changing from one bariatric procedure type to a different procedure type. Conversion procedures included conversion to SG, RYGB, distalization, BPD, BPD-DS, SADI-DS, OAGB, and other unlisted conversions. Revision procedures involve modifications or corrective procedures to address complications or inadequate response while maintaining the same general procedure type. Revision procedures included RYGB revision, SG revision, gastric pouch or stoma plication, adjustable gastric band (AGB) revision, Roux limb lengthening, gastrectomy, AGB placement over RYGB, SADI-DS revision, BPD/BPD-DS revision, and other unlisted revisions. Surgical approach (laparoscopic, open, or endoscopic), drain placement, and length of stay were recorded.

## Outcome Variables

The primary outcomes included characterizing 5-year trends in procedure delivery. Secondary outcomes included complications, such as anastomotic leak, postoperative bleeding, reoperation, reintervention, readmission, cardiac complications, pneumonia, acute kidney injury, venous thromboembolism, and serious complications (composite outcome defined as any of the above complications or death). Mortality was defined as death within 30 days of the index operation.

### Statistical Analysis

Categorical variables were expressed as frequencies and percentages while continuous variables were presented as means with standard deviations. Temporal trends across years were evaluated using chi-square tests for categorical variables and linear regression for continuous variables. A *p*-value of < 0.05 was considered statistically significant. All statistical analyses were performed using STATA 17 statistical software (StataCorp, College Station, TX, USA).

## Results

### Baseline Demographics and Comorbidities

Among primary, revisional, and conversion procedures, female predominance increased from 81.73% (*N* = 137,768) to 82.48% (*N* = 146,642), whereas mean age decreased slightly from 44.17 ± 11.86 years to 43.55 ± 11.95 years from 2020 to 2024, respectively (both *p* < 0.0001). Mean body mass index remained stable at 44.66 ± 7.98 kg/m² in 2020 and 44.75 ± 8.34 kg/m². Racial and ethnic diversity increased significantly within the study time frame. White race predominance declined from 67.38% (*N* = 113,585) to 60.09% (*N* = 106,836), Black patients increased from 19.68% (33,178) to 20.54% (36,516), and American Indian, Alaska Native, Asian, and other races rose from 12.94% (21,805) to 19.37% (34,437) (*p* < 0.0001).

Patient medical complexity increased across several domains from 2020 to 2024. The proportion of patients in ASA Class III or above increased from 79.77% (*N* = 134,459) to 82.45% (*N* = 146,594) (*p* < 0.0001). Sleep apnea increased from 36.36% (*N* = 61,294) to 37.63% (*N* = 66,901), hyperlipidemia from 22.17% (*N* = 37,366) to 23.28% (*N* = 41,388), history of venous thromboembolism from 2.61% (*N* = 4,401) to 3.05% (*N* = 5,417), and venous stasis from 0.71% (*N* = 1,189) to 1.02% (*N* = 1,805). The use of therapeutic anticoagulation at the time of bariatric surgery increased from 2.89% (*N* = 4,878) to 3.36% (*N* = 5,980). Preoperative gastroesophageal reflux disease (GERD) increased from 32.58% (*N* = 54,926) to 34.63% (*N* = 61,573) (all *p* < 0.0001). Of note, the proportion of patients that were smokers at the time of surgery decreased from 6.87% (*N* = 11,586) to 5.72% (*N* = 10,165) (*p* < 0.0001) (Table 1).

**Table 1:** Baseline Patient Demographics and Comorbidities, 2020-2024

Characteristic	2020	2021	2022	2023	2024	*P*- Value
Female	137,768 (81.7)	175,416 (83.0)	191,494 (83.0)	180,099 (82.6)	146,642 (82.5)	< 0.0001
Age	44.2 ± 11.9	43.6 ± 11.6	43.8 ± 11.7	43.7 ± 11.9	43.6 ± 12.0	< 0.0001
BMI	44.7 ± 8.0	44.7 ± 7.9	44.7 ± 7.9	44.7 ± 8.1	44.8 ± 8.3	0.001
Race						
White	113,585 (67.4)	136,415 (64.6)	147,011 (63.7)	135,152 (62.0)	106,836 (60.1)	< 0.0001
Black	33,178 (19.7)	43,297 (20.5)	47,858 (20.7)	44,330 (20.3)	36,516 (20.5)	
Other	21,805 (12.9)	31,542 (14.9)	35,838 (15.5)	38,470 (17.7)	34,437 (19.4)	
Comorbidities						
ASA Class ≥ III	134,459 (79.8)	170,197 (80.6)	188,004 (81.5)	178,526 (81.9)	146,594 (82.5)	< 0.0001
Smoker	11,586 (6.9)	13,692 (6.5)	13,856 (6.0)	13,102 (6.0)	10,165 (5.7)	< 0.0001
GERD	54,926 (32.6)	70,003 (33.1)	77,473 (33.6)	73,468 (33.7)	61,573 (34.6)	< 0.0001
Diabetes	38,074 (22.6)	45,149 (21.4)	51,912 (22.5)	49,717 (22.8)	39,341 (22.1)	< 0.0001
Hypertension	N/A	90,279 (42.7)	100,903 (43.7)	95,060 (43.6)	75,919 (42.7)	< 0.0001
Sleep apnea	61,294 (36.4)	74,324 (35.2)	82,532 (35.8)	80,879 (37.1)	66,901 (37.6)	< 0.0001
Hyperlipidemia	37,366 (22.2)	45,214 (21.4)	52,204 (22.6)	50,867 (23.3)	41,388 (23.3)	< 0.0001
COPD	2,064 (1.22)	2,355 (1.11)	2,424 (1.05)	2,653 (1.22)	2,092 (1.18)	< 0.0001
Renal Insufficiency	941 (0.56)	1,053 (0.50)	1,046 (0.45)	1,165 (0.53)	910 (0.51)	< 0.0001
Dialysis	508 (0.30)	584 (0.28)	624 (0.27)	672 (0.31)	516 (0.29)	0.106
History of VTE	4,401 (2.6)	5,409 (2.6)	6,331 (2.7)	6,388 (2.9)	5,417 (3.05)	< 0.0001
Venous stasis	1,189 (0.71)	1,229 (0.58)	1,772 (0.77)	1,878 (0.86)	1,805 (1.02)	< 0.0001
Therapeutic anticoagulation	4,878 (2.89)	5,670 (2.68)	7,046 (3.05)	6,904 (3.17)	5,980 (3.36)	< 0.0001
History of MI	1,775 (1.05)	2,021 (0.96)	2,299 (1.0)	2,264 (1.0)	1,868 (1.1)	< 0.0001
Prior cardiac intervention	1,530 (0.91)	1,673 (0.79)	1,699 (0.74)	1,590 (0.73)	1,307 (0.74)	< 0.0001
Prior PCI/stent	2,515 (1.5)	2,552 (1.2)	2,796 (1.2)	2,525 (1.2)	1,937 (1.1)	< 0.0001
Immunosuppressive therapy	N/A	4,745 (2.3)	5,509 (2.4)	5,525 (2.5)	4,658 (2.6)	< 0.0001

*BMI* Body Mass Index, *ASA* American Society of Anesthesiologists, *GERD* Gastroesophageal reflux disease, *COPD* Chronic Obstructive Pulmonary Disease, *VTE* Venous Thromboembolic Event, *PCI* Percutaneous Coronary Intervention

## Overall Procedure Distribution

**Fig. 1:**
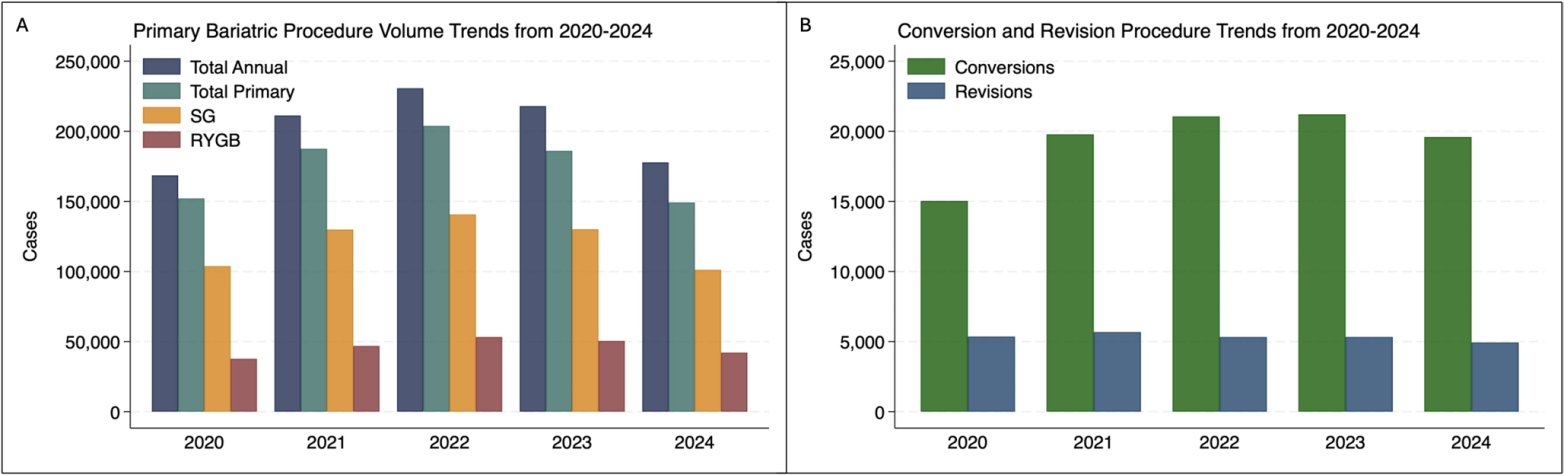
Bariatric Surgery Volume Trends, 2020–2024 from Metabolic and Bariatric Surgery Accreditation and Quality Improvement Program (MBSAQIP) database. (A) Total annual volume, primary procedures, sleeve gastrectomy (SG), and Roux-en-Y gastric bypass (RYGB). (B) Secondary procedures including conversions and revisions.

Total annual procedure volumes increased from 168,568 cases in 2020 to a peak of 230,707 in 2022, followed by a decline to 217,952 in 2023 and 177,789 in 2024, representing a 23% decrease from the 2022 peak and a 5% increase overall from 2020 to 2024. The number of MBSAQIP-accredited centers increased from 885 in 2020 to 902 in 2021 (+ 1.9%), 924 in 2022 (+ 2.4%), 961 in 2023 (+ 4.0%), and 959 in 2024 (− 0.2%), representing an overall 8.4% increase in participating centers over the study period [10]. Despite this expansion in accredited centers, average case volume per center declined from 190 cases per center in 2020 to 234 in 2021, 250 cases per center at the 2022 peak, 227 in 2023, and 185 cases per center in 2024 (− 26% from peak), falling below 2020 pandemic-era levels and indicating that declining volumes were not offset by center expansion [10, 11]. Primary procedures decreased throughout the study period, comprising 90.2% (*N* = 152,090) of all MBS procedures in 2020, 88.8% (*N* = 187,519) in 2021, 88.4% (*N* = 203,832) in 2022, 85.4% (*N* = 186,030) in 2023, and 83.9% (*N* = 149,191) in 2024 (*p* < 0.0001). Conversion procedures increased significantly from 8.9% (*N* = 15,031) in 2020 to 11.0% (*N* = 19,592) in 2024 (*p* < 0.0001). Revision procedures comprised 3.2% (*N* = 5,356) of MBS in 2020, 2.7% (*N* = 5,679) in 2021, 2.3% (*N* = 5,326) in 2022, 2.4% (*N* = 5,327) in 2023, and 2.8% (*N* = 4,935) in 2024 (*p* < 0.0001). Laparoscopic and robotic approach for primary surgery, conversions, and revisions increased from 98.8% (*N* = 166,506) to 99.0% (*N* = 176,017), while open procedures declined from 0.31% (*N* = 520) to 0.11% (*N* = 204) (*p* < 0.0001). Drain placement decreased from 9.9% (*N* = 16,681) to 5.6% (*N* = 9,880) (*p* < 0.001) (Fig. 1; Supplemental Figure 1; Table 2).

**Table 2:** Procedure Distribution and Volume Trends, 2020-2024

Procedure type	2020	2021	2022	2023	2024	*P*- Value
Total annual volume	168,568	211,254	230,707	217,952	177,789	
Primary procedures	152,090 (90.2)	187,519 (88.8)	203,832 (88.4)	186,030 (85.4)	149,191 (83.9)	< 0.0001
SG	103,782 (73.4)	129,881 (73.5)	140,605 (72.5)	130,123 (72.1)	101,145 (70.6)	
RYGB	37,690 (26.6)	46,857 (26.5)	53,242 (27.5)	50,435 (27.9)	42,114 (29.4)	
BPD-DS	1,920 (1.3)	2,514 (1.4)	3,368 (1.65)	3,043 (1.59)	2,249 (1.47)	
OAGB	1,338 (0.9)	944 (0.51)	903 (0.44)	457 (0.24)	232 (0.15)	
ESG	771 (0.52)	677 (0.36)	727 (0.36)	581 (0.30)	364 (0.24)	
SADI-DS	488 (0.33)	842 (0.45)	1, 338 (0.65)	1, 963 (1.0)	2,670 (1.7)	
Intragastric balloon	346 (0.23)	430 (0.23)	440 (0.22)	229 (0.12)	96 (0.06)	
BPD	153 (0.10)	247 (0.13)	269 (0.13)	62 (0.03)	8 (0.01)	
Other, not listed	5,602 (3.8)	5,127 (2.8)	4,278 (2.1)	1,100 (0.57)	313 (0.20)	
Conversion Procedures	15,031 (8.9)	19,780 (9.4)	21,057 (9.2)	21,209 (9.7)	19,592 (11.0)	< 0.0001
RYGB	8, 621 (57.4)	12, 623 (63.8)	14,042 (66.7)	14, 780 (69.7)	14, 068 (71.8)	
SG	4,252 (28.3)	4,454 (22.5)	4,041 (19.2)	3,268 (15.4)	2,216 (11.3)	
BPD-DS	759 (5.0)	1,028 (5.2)	1,115 (5.3)	1,017 (4.8)	907 (4.6)	
Distalization	496 (3.3)	572 (2.9)	537 (2.6)	522 (2.5)	537 (2.7)	
SADI-DS	391 (2.6)	542 (2.7)	782 (3.7)	1,105 (5.2)	1,209 (6.2)	
OAGB	148 (0.98)	130 (0.66)	163 (0.77)	142 (0.67)	190 (0.97)	
BPD	18 (0.12)	24 (0.12)	26 (0.12)	22 (0.l0)	26 (0.13)	
Other, not listed	356 (2.4)	407 (2.1)	351 (1.7)	353 (1.7)	439 (2.2)	
Revision	5,356 (3.2)	5,679 (2.7)	5,326 (2.3)	5,327 (2.4)	4,935 (2.8)	< 0.0001
RYGB revision	2,469 (46.1)	2,779 (48.9)	2,663 (50.0)	2,755 (51.7)	2,670 (54.1)	
SG revision	658 (12.3)	878 (15.5)	870 (16.3)	812 (15.2)	662 (13.4)	
Gastric pouch/stoma plication	479 (8.9)	664 (11.7)	744 (14.0)	741 (13.9)	596 (12.1)	
AGB revision	652 (12.2)	411 (7.2)	339 (6.4)	171 (3.2)	128 (2.6)	
Roux limb lengthening	154 (2.9)	176 (3.1)	231 (4.3)	313 (5.9)	318 (6.4)	
Gastrectomy	164 (3.1)	104 (1.83)	49 (0.92)	83 (1.6)	69 (1.4)	
AGB over RYGB	89 (1.7)	51 (0.90)	61 (1.15)	42 (0.79)	49 (0.99)	
SADI-DS revision	22 (0.41)	27 (0.48)	30 (0.56)	40 (0.75)	70 (1.42)	
BPD/BPD-DS revision	62 (1.16)	80 (1.41)	73 (1.37)	79 (1.48)	73 (1.48)	
Other/Unlisted	607 (11.3)	509 (9.0)	266 (5.0)	291 (5.5)	300 (6.1)	

*SG* Sleeve Gastrectomy, *RYGB* Roux-en-Y Gastric Bypass, *BPD-DS* Biliopancreatic Diversion with Duodenal Switch, *OAGB* One Anastomosis Gastric Bypass, *ESG* Endoscopic Sleeve Gastroplasty, *SADI-DS* Single Anastomosis Duodeno-Ileal Bypass with Sleeve Gastrectomy, *BPD* Biliopancreatic Diversion, *AGB* Adjustable Gastric Banding

### Primary Procedures

Sleeve gastrectomy remained the most common primary procedure throughout the study period but declined in proportion of delivery from 103,782 cases (73.4% of primary procedures) in 2020 to 129,881 cases (73.5%) in 2021, 140,605 cases (72.5%) in 2022, 130,123 cases (72.1%) in 2023, and 101,145 cases (70.6%) in 2024 (*p* < 0.0001). Roux-en-Y gastric bypass increased as a proportion of primary procedures from 26.6% (*N* = 37,690) in 2020 to 26.5% (*N* = 26.5) in 2021, 27.5% (*N* = 27.5) in 2022, 27.9% (*N* = 50,435) in 2023, and 29.4% (*N* = 42,114) in 2024, with absolute case volumes peaking in 2022 (*p* < 0.0001). Single-anastomosis duodeno-ileal bypass/loop duodenal switch increased from 0.29% (*N* = 488) in 2020 to 1.50% (*N* = 2,670) in 2024. Biliopancreatic diversion with duodenal switch increased from 1.14% (*N* = 1,920) to 1.26% (*N* = 2,249). (Fig. 1; Table 2).

### Conversion Procedures

**Fig. 2:**
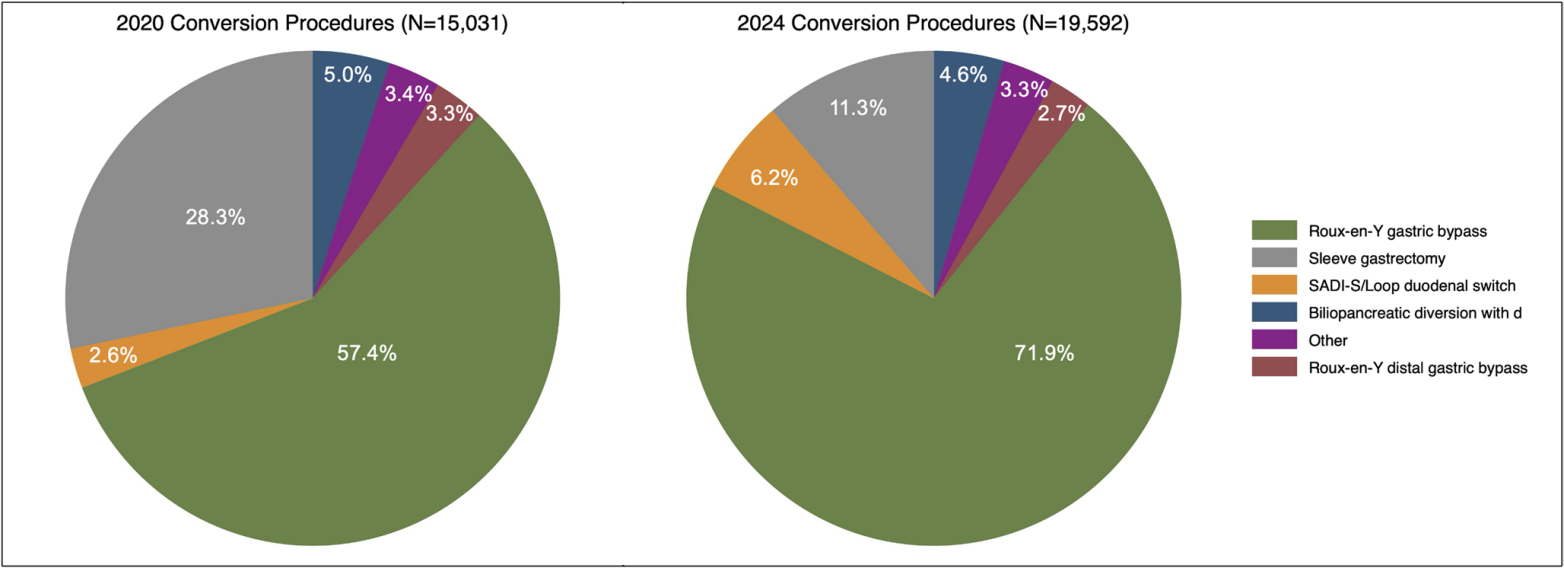
Conversion Procedure Distribution, 2020 and 2024 from Metabolic and Bariatric Surgery Accreditation and Quality Improvement Program (MBSAQIP) database. Left: 2020 conversion procedures (N = 15,031). Right: 2024 conversion procedures (N = 19,592).

Among conversion procedures, conversion to Roux-en-Y gastric bypass was the most common target procedure, increasing from 57.35% (*N* = 8,621) of conversions in 2020 to 63.80% (*N* = 12,623) in 2021, 66.70% (*N* = 14,042) in 2022, 69.70% (*N* = 14,780) in 2023, and 71.80% (*N* = 14,068) in 2024. Conversions to SG decreased from 28.30% (*N* = 4,252) in 2020 to 22.50% (*N* = 4,454) in 2021, 19.20% (*N* = 4,041) in 2022, 15.40% (*N* = 3,268) in 2023, and 11.30% (*N* = 2,216) in 2024. Conversions to BPD-DS decreased from 5.00% (*N* = 759) in 2020 to 4.60% (*N* = 907) in 2024. Conversion to distal RYGB remained stable at 3.30% (*N* = 496) in 2020 to 2.70% (*N* = 537) in 2024. Conversion to BPD remained rare at 0.12% (*N* = 18) in 2020 to 0.13% (*N* = 26) in 2024 (Fig. 2; Table 2).

### Revision Procedures

RYGB revision was the most common revisional procedure at 46.10% (*N* = 2,469) in 2020, increasing to 54.10% (*N* = 2,670) in 2024. Sleeve gastrectomy revision (re-sleeve) comprised 12.29% (*N* = 658) in 2020 and 13.41% (*N* = 662) in 2024. Gastric pouch or stoma plication increased from 8.94% (*N* = 479) to 12.08% (*N* = 596). Adjustable gastric band port and/or tubing revision decreased from 12.17% (*N* = 652) to 2.59% (*N* = 128). Roux-en-Y limb lengthening increased from 2.88% (*N* = 154) to 6.44% (*N* = 318) (all *p* < 0.0001) (Table 2, Fig. 1).

### Perioperative Outcomes

Mean length of stay declined from 1.44 ± 1.42 days in 2020 to 1.28 ± 1.37 days in 2024 (*p* < 0.001). However, thirty-day readmission rates increased from 3.22% (*N* = 5,435) to 3.58% (*N* = 6,363) (*p* < 0.0001). Serious complication rate decreased from 3.39% (*N* = 5,710) in 2020 to 3.10% (*N* = 5,520) in 2024. Reoperation within 30 days decreased from 1.20% (*N* = 2,031) to 1.10% (*N* = 1,962). Rates of anastomotic or staple line leak (0.33% to 0.28%), re-intervention (0.87% to 0.90%), and bleeding (1.1% to 0.90%) remained relatively stable from 2020 to 2024. Thirty-day mortality remained stable at 0.08% (*N* = 134) in 2020 and 0.06% (*N* = 106) in 2024 (Table 3).

**Table 3:** Operative Characteristics and Perioperative Outcomes, 2020-2024

Characteristic	2020	2021	2022	2023	2024	*P*-Value
Surgical approach						
Laparoscopic	166,506 (98.8)	208,797 (98.8)	228,162 (98.9)	215,656 (99.0)	176,017 (99.0)	< 0.0001
Open	520 (0.31)	336 (0.16)	279 (0.12)	284 (0.3)	204 (0.11)	< 0.0001
Endoscopic	1,542 (0.91)	2,121 (1.0)	2,266 (0.98)	2,012 (0.92)	1,568 (0.88)	< 0.0001
Drain placement	151,887 (90.1)	193,573 (91.6)	214,741 (93.1)	204,907 (94.0)	167,909 (94.4)	< 0.0001
Length of stay	1.44 ± 1.4	1.35 ± 1.4	1.29 ± 1.3	1.28 ± 1.3	1.28 ± 1.4	< 0.0001
Complications						
Leak	552 (0.33)	685 (0.32)	672 (0.29)	621 (0.28)	505 (0.28)	0.017
Bleed	1,769 (1.1)	2,081 (0.99)	2,203 (0.95)	2,044 (0.94)	1,608 (0.90)	< 0.0001
Reoperations	2,031 (1.20)	2,508 (1.19)	2,588 (1.12)	2,350 (1.08)	1,962 (1.10)	< 0.0001
Reintervention	1,460 (0.87)	1,953 (0.92)	1,877 (0.81)	1,794 (0.82)	1,596 (0.90)	< 0.0001
Readmission	5,435 (3.2)	7,068 (3.4)	7,500 (3.3)	7,317 (3.4)	6,363 (3.6)	< 0.0001
Cardiac event	196 (0.12)	243 (0.12)	282 (0.12)	280 (0.13)	222 (0.12)	0.707
Pneumonia	397 (0.24)	477 (0.23)	475 (0.21)	483 (0.22)	422 (0.24)	0.207
Acute kidney injury	202 (0.12)	206 (0.10)	241 (0.10)	253 (0.12)	188 (0.11)	0.200
Venous thromboembolic events	663 (0.39)	784 (0.37)	858 (0.37)	783 (0.36)	651 (0.37)	0.527
Serious complications	5,710 (3.39)	7,020 (3.32)	7,121 (3.09)	6,620 (3.04)	5,520 (3.10)	< 0.0001
Mortality	134 (0.08)	169 (0.08)	193 (0.08)	185 (0.08)	106 (0.06)	0.039

### Adjusted Analysis of Serious Complications

After adjustment for patient demographics, comorbidities, functional status, procedure type, and operative length, later operative year remained independently associated with lower odds of serious complication relative to 2020 (2022: aOR 0.92, 95% CI 0.89 to 0.95; 2023: aOR 0.88, 95% CI 0.85 to 0.92; 2024: aOR 0.87, 95% CI 0.84 to 0.90; all *p* < 0.001). Independent predictors of serious complication included age (aOR 1.08 per decade), male sex (aOR 1.10), insulin-dependent diabetes (aOR 1.11), GERD (aOR 1.25), bypass (aOR 1.45), revisional procedure (aOR 1.45), partial functional dependence (aOR 1.60), total functional dependence (aOR 2.27), renal insufficiency (aOR 1.99), therapeutic anticoagulation (aOR 1.63), history of myocardial infarction (aOR 1.44), and history of VTE (aOR 1.53). The model demonstrated modest discrimination (AUC 0.669) with adequate calibration (Brier score 0.030; Spiegelhalter z = 0.35, *p* = 0.36) (Supplemental Table 1).

## Discussion

This analysis of over one million bariatric procedures from 2020 to 2024 demonstrates significant volume decline, particularly when adjusted for growth in accredited bariatric centers, with per-center volumes falling to pandemic-era levels by 2024. Sleeve gastrectomy (SG) utilization decreased while Roux-en-Y gastric bypass (RYGB) and conversion procedures increased significantly. Patient medical complexity increased modestly across several comorbidity domains, yet serious complication rates declined while mortality remained stable. The protective signal for later operative year persisted after multivariable adjustment for demographics, comorbidities, functional status, and procedure type, supporting a true improvement in perioperative safety rather than a shift in case mix alone. While individual shifts in comorbidities were modest in absolute terms, several of the variables that increased over the study period, including therapeutic anticoagulation (aOR 1.63), renal insufficiency (aOR 1.99), history of VTE (aOR 1.53), and partial functional dependence (aOR 1.60), were among the strongest independent predictors of serious complication, suggesting that the cumulative shift in case mix is clinically meaningful rather than statistical noise. These findings suggest changing practice patterns and improvements in quality of care during a period of overall declining volumes.

Total volumes declined 23% from the 2022 peak of 230,707 procedures to 177,789 in 2024, with an 18% reduction from 2023 to 2024. Although 2024 absolute volumes remain modestly above 2020 levels (168,568 procedures), per-center volumes have fallen to pandemic-era levels when accounting for the concurrent expansion in accredited centers. National volumes had rebounded from approximately 199,000 procedures in 2020 to 280,000 in 2022 [12], yet the present analysis reveals this recovery was short-lived. More concerning, when accounting for the 4.4% growth in MBSAQIP-accredited centers (from 885 in 2020 to 924 in 2022) [11], per-center volume declined from 250 cases at the 2022 peak to approximately 193 cases per center in 2024, a level not seen since the height of the COVID-19 pandemic (190 cases per center in 2020 The timing of this decline coincides with rapid anti-obesity medication (AOM) adoption, and recent ecologic analysis has shown a 25.6% decrease in bariatric surgery rates alongside a more than two-fold increase in GLP-1 RA prescriptions from 2022 to 2023 [9]. The MBSAQIP database does not permit causal inference between these trends, and several alternative explanations warrant consideration. Post-pandemic recovery dynamics, evolving insurance coverage and prior authorization requirements, shifting referral patterns from primary care toward medical weight management, and workforce or capacity constraints at accredited centers may each contribute to the observed volume changes. The contribution of GLP-1 RA adoption is plausible but cannot be quantified from registry data alone. While bariatric surgery currently achieves superior weight loss compared to GLP-1 RAs [13], patient preference for, and access to, non-surgical options may be contributing to these trends. Notably, fewer than 6% of eligible patients receive either surgical or medical intervention [9], suggesting that the need for obesity treatment has not diminished.

The shift from SG to RYGB likely reflects accumulating long-term outcome data favoring RYGB superiority for durable weight loss and metabolic comorbidity resolution. RYGB demonstrates superior outcomes, with 14% greater excess weight loss at 5 years, 72% higher type 2 diabetes remission rates, and better sustained hypertension control compared to SG [14]. Notably, gastroesophageal reflux disease (GERD) has emerged as a major limitation of SG, with de novo GERD developing in 21–32% of patients based on definitions and approximately 4% requiring conversion to RYGB for medically refractory reflux [15–18]. The disproportionate decline in SG may also reflect lower BMI patients increasingly selecting GLP-1 RA therapy, as these medications are approved at lower BMI thresholds than surgery, creating overlapping eligibility.

Beyond primary procedure selection, the case mix has shifted substantially toward conversion and revision procedures. Conversion to RYGB has become the dominant conversion procedure, increasing from 57% to 72% of all conversions with absolute volumes rising 63%, while conversions to SG declined by more than half. Among revision procedures, RYGB revision and Roux limb lengthening both increased substantially, while adjustable gastric band revisions declined sharply, reflecting the near abandonment of this as a primary procedure. GERD represents 30–55% of conversion indications [19, 20], and the increasing proportion of conversions to RYGB reflects the growing recognition of this procedure as an effective solution for reflux after SG. These data demonstrate a fundamental shift in bariatric practice toward more technically demanding revision procedures and complication management, moving away from primary operations in treatment-naive patients.

Despite declining surgical volumes, obesity remains undertreated, with fewer than 6% of eligible patients receiving either bariatric surgery or AOMs. Access to GLP-1 receptor agonists remains limited, with only 3% of eligible patients able to obtain these medications due to insurance coverage restrictions, high financial costs, and supply constraints [21]. The trajectory of bariatric surgery volumes over the next 5–10 years remains uncertain and likely depends on GLP-1 access and long-term efficacy. Continued surgical volume decline is possible with improved medication access and adherence, though volumes may stabilize or gradually recover. Preoperative GLP-1 receptor agonist use among bariatric surgery patients has tripled from 2018 to 2023 (5% to over 15%), with rates of AOM use as high as 43% among those with class III obesity [22, 23], demonstrating that surgery is increasingly serving patients who have attempted medical management first. Given high discontinuation rates and substantial weight recurrence after stopping AOMs, many patients who begin with medical management may eventually seek surgery [24, 25]. The growing population initiating these medications also increases overall exposure to metabolic and bariatric medicine, which may ultimately increase the surgical candidate pool. The future may involve balancing roles, with AOMs as first-line therapy and surgery reserved for patients requiring more aggressive intervention, medication intolerance, or durable weight loss without lifelong medication dependence.

The observed volume trends and shifting case mix have important implications for bariatric surgery training. Prior to the current decline, mean RYGB cases per bariatric fellow had already fallen from 91.1 to 52.6 cases (2012–2019) [26], below the 75–100 case threshold considered to be needed for RYGB competency [27]. Our analysis demonstrates this challenge has intensified, with per-center volumes declining to 193 cases by 2024, approaching minimum MBSAQIP accreditation thresholds. Simultaneously, conversion procedures, which typically require substantially more advanced technical skills than primary operations, increased from 8.92% to 11.02% of total cases, and prior data show revisional cases doubled from 8 to 19 per fellow [28]. This shift highlights the need for fellowship training that incorporates management of altered anatomy, complex adhesiolysis, and revisional procedures in addition to primary operations. As AOM use expands and surgical volumes decline, bariatric surgeons would also benefit from broader competencies encompassing medical and surgical obesity management, including perioperative medication management, patient selection for multimodal approaches, and obesity medicine accreditation [9]. Fellowship programs should ensure adequate exposure to revisional and conversion procedures through extended training, dedicated rotations, or simulation-based education.

Two additional trends warrant discussion. First, substantial demographic shifts occurred during the study period, with the proportion of White patients declining from 67.38% to 60.09% and the American Indian, Alaska Native, Asian, and other racial category increasing from 12.94% to 19.37% (both *p* < 0.0001). This increased diversity suggests progress toward equitable access for historically underserved populations, though significant disparities persist and continued efforts are needed [9]. Second, mean length of stay decreased from 1.44 to 1.28 days while 30-day readmission rates increased from 3.22% to 3.58% (both *p* < 0.0001), suggesting a potential tradeoff between early discharge protocols and readmission risk, though this relationship has not been established in the literature and needs further investigation [29, 30].

This study has several limitations. The use of the database and retrospective design limits assessment of granular clinical details like preoperative laboratory values, medications, and long-term outcomes beyond 30 days. Surgeon-level experience and volume, as well as geographic variation in practice patterns, insurance requirements, and patient demographics, are not captured and likely influence observed trends. The very large sample size can also produce statistically significant associations at small effect sizes, an intrinsic limitation of registry-based research, and the temporal questions addressed here are not feasible to study in a randomized design. Despite these limitations, the large sample size and standardized data collection offer valuable insights into current bariatric surgery trends.

## Conclusion

This analysis of over one million bariatric procedures demonstrates evolving practice patterns in modern bariatric surgery, including a 23% reduction in total volumes from the 2022 peak of 230,707 procedures to 177,789 in 2024, with 2024 volumes still exceeding 2020 levels. When adjusted for growth in accredited centers, per-center volumes declined from 250 cases at the 2022 peak to 193 cases in 2024, falling to pandemic-era levels. Sleeve gastrectomy volumes declined 26% while conversion procedures increased from 8.92% to 11.02% of total cases (+ 30% absolute increase) relative to primary sleeve gastrectomy. Despite rising patient complexity, serious complication rates declined and mortality remained stable, reflecting centralization to accredited centers and learning curve maturation. These findings demonstrate the evolving role of bariatric surgery in a landscape shaped by pharmacologic alternatives, post-pandemic recovery dynamics, and persistent access barriers, though the relative contribution of each driver cannot be determined from registry data.

## Supplementary Information

Below is the link to the electronic supplementary material.

Supplementary Material 1

Supplementary Material 2

## Data Availability

The data used analyzed here has been compiled from the Metabolic and Bariatric Surgery Accreditation Quality Improvement Program (MBSAQIP) database.
