# Supplementary figures and images for: Declining Bariatric Surgery Volumes and Shifting Practice Patterns: A Five-Year Analysis of Over One Million Procedures
Source: Obes Surg. 2026 Jul 15;36(8):4065–74. doi: 10.1007/s11695-026-08795-y (PMC13429527; doi:10.1007/s11695-026-08795-y)

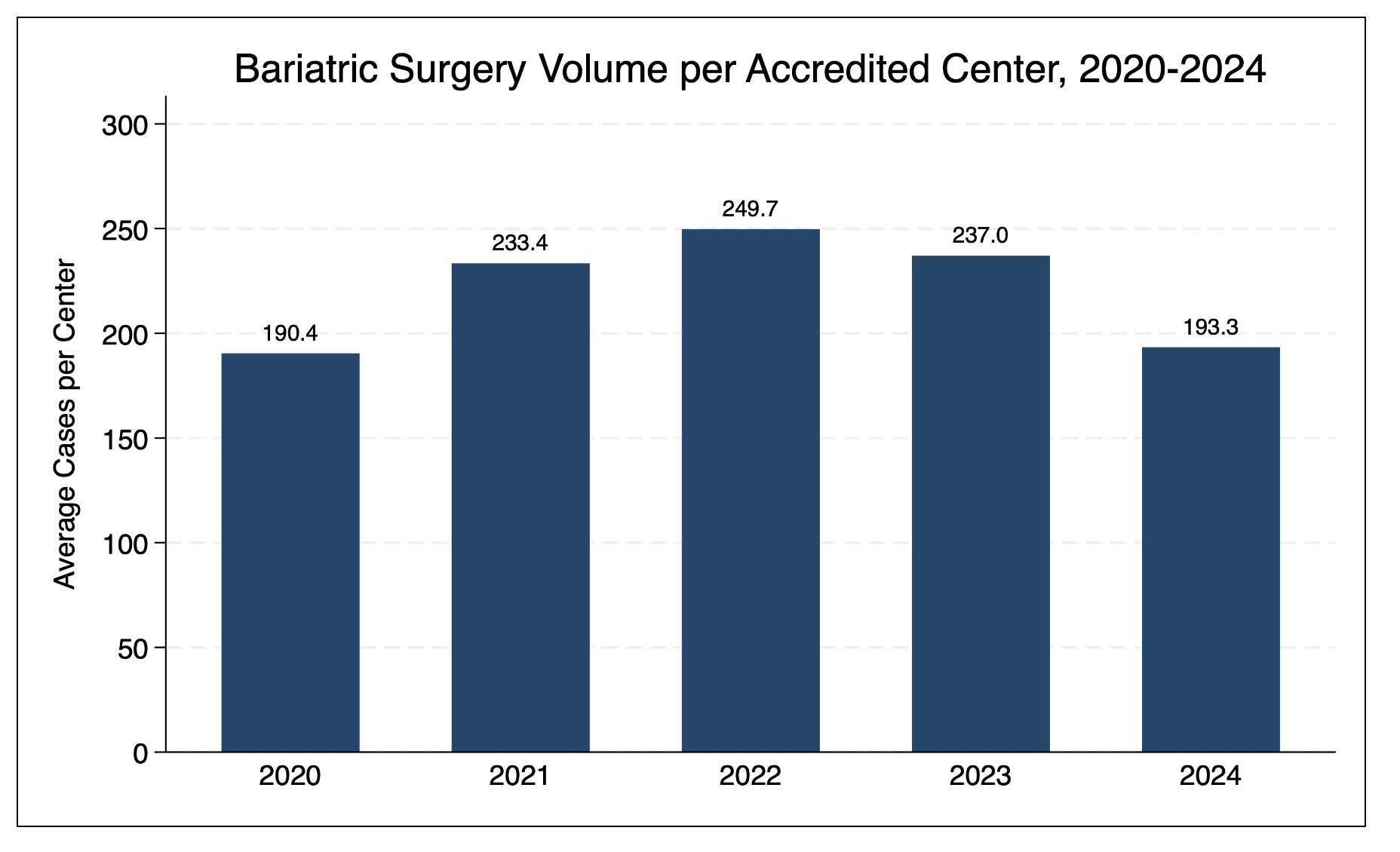

Supplement: Supplementary file 1 — Supplementary Material 1 [file 11695_2026_8795_MOESM1_ESM.png]
