# Supplementary material for: Declining Bariatric Surgery Volumes and Shifting Practice Patterns: A Five-Year Analysis of Over One Million Procedures
Source: Obes Surg. 2026 Jul 15;36(8):4065–74. doi: 10.1007/s11695-026-08795-y (PMC13429527; doi:10.1007/s11695-026-08795-y)
Supplement: Supplementary file 2 — Supplementary Material 2 [file 11695_2026_8795_MOESM2_ESM.docx]

**Supplemental Table I.** Multivariable logistic regression for serious complication following bariatric surgery, MBSAQIP 2020–2024.

| **Variable** | **Adjusted OR** | **95% CI** | **p-value** |
| --- | --- | --- | --- |
| Age (per 10 years) | 1.08 | 1.07 to 1.09 | <0.001 |
| BMI (per 5 kg/m²) | 0.99 | 0.98 to 1.00 | 0.007 |
| Female sex | 0.90 | 0.88 to 0.94 | <0.001 |
| Diabetes, non-insulin dependent | 0.99 | 0.96 to 1.03 | 0.640 |
| Diabetes, insulin dependent | 1.11 | 1.06 to 1.17 | <0.001 |
| Operative length (per minute) | 1.004 | 1.004 to 1.004 | <0.001 |
| GERD | 1.25 | 1.21 to 1.28 | <0.001 |
| Operative year 2022 (ref: 2020) | 0.92 | 0.89 to 0.95 | <0.001 |
| Operative year 2023 (ref: 2020) | 0.88 | 0.85 to 0.92 | <0.001 |
| Operative year 2024 (ref: 2020) | 0.87 | 0.84 to 0.90 | <0.001 |
| Hypertension | 1.13 | 1.10 to 1.16 | <0.001 |
| Functional status, partially dependent | 1.60 | 1.41 to 1.82 | <0.001 |
| Functional status, totally dependent | 2.27 | 1.37 to 3.76 | 0.002 |
| Bypass procedure | 1.45 | 1.41 to 1.49 | <0.001 |
| History of VTE | 1.53 | 1.45 to 1.63 | <0.001 |
| Race category 1 | 1.23 | 1.20 to 1.27 | <0.001 |
| Race category 2 | 0.95 | 0.91 to 0.98 | 0.006 |
| Renal insufficiency | 1.99 | 1.77 to 2.23 | <0.001 |
| Therapeutic anticoagulation | 1.63 | 1.54 to 1.72 | <0.001 |
| History of myocardial infarction | 1.44 | 1.32 to 1.58 | <0.001 |
| Procedure type, initial (ref: conversion) | 0.71 | 0.68 to 0.74 | <0.001 |
| Procedure type, revision (ref: conversion) | 1.45 | 1.36 to 1.55 | <0.001 |

Model discrimination: AUC 0.669. Calibration: Brier score 0.030; Spiegelhalter z = 0.35, p = 0.36. Reference categories: operative year 2020, conversion procedure. Race categories follow MBSAQIP coding.
